# Transparent PEDOT:PSS/PDMS Leaf Tattoos for Multiplexed Plant Health Monitoring and Energy Harvesting

**DOI:** 10.3390/bios15120805

**Published:** 2025-12-09

**Authors:** Antonio Ruiz-Gonzalez, Harriet Kempson, Jim Haseloff

**Affiliations:** Department of Plant Sciences, University of Cambridge, Downing St., Cambridge CB2 3EA, UK; a.gonzalez.16@ucl.ac.uk (A.R.-G.);

**Keywords:** wearable biosensors, plant health, tattoo electrode

## Abstract

The development of non-invasive sensors for individualised plant monitoring has become essential in smart farming to increase crop production. However current approaches are focused on the measurement of soil parameters instead, which cannot provide direct information about plant health. Moreover, equipment used for the direct monitoring of plant health are costly with complex operation, hindering their use by the wider community of farmers. This work reports for the first time the development of a flexible and highly transparent sensor, based on thin conductive PEDOT:PSS/PDMS hybrid films directly deposited onto leaves. The films were fabricated by aerosol deposition and could operate under two different modes. The first mode is used for the determination of plant dryness and concentration of ions. The second mode is used as a triboelectric generator to generate up to 7.2 µW cm^−2^ electrical power through the friction of the sensors with a leaf. The device was assembled using a low-cost (GBP < 70) microcontroller incorporating environmental sensors, and an intuitive interface was designed for operation. The final sensor could determine the ionic strength at the millimolar level by means of the impedance of electrodes. This performance allowed the study of differences in ionic content and water availability in tomato leaves during day–night cycles. The high stability of the sensors also allowed the long-term monitoring of plant health. Using this technology, a decrease in the leaf ionic strength due to the lack of electrolytes was observed after watering with deionised water for 2 days. Upon supplementation with fertiliser, the recorded ionic strength and leaf water content were similar to the original values prior to the use of DI water, demonstrating the applicability of the device in the early detection of stress factors that could decrease crop production.

## 1. Introduction

The development of technologies for direct plant health monitoring has become a crucial aspect in crop science research. This technology could be used for the individualised monitoring of plant health, reducing production losses due to biological threats, such as pathogens and pests, and abiotic stresses including drought, heat, or soil acidity [1]. As such, it could be used to tackle the 70% increase in food production needed to feed the global population by 2050 [2,3]. However, currently available technologies on the market are relatively expensive and cannot be used for the individualised assessment of crops. Moreover, there is a lack of academic work focused on non-invasive monitoring of plant health.

Within the past few years the development of wearable sensors for human health monitoring has experienced an unprecedented development, enabling the non-invasive monitoring of key biomarkers such as glucose [4], lactose [5], and electrolytes such as potassium or sodium ions [6], among others. Specifically, the use of skin-inspired materials as sensing electrodes could enable multiplexed monitoring using non-invasive and flexible materials [7]. However, despite the fast development of these technologies in healthcare, to date, only a limited number of these discoveries have been translated to the field of plant research [1]. The development of miniaturised sensors for plant monitoring has traditionally been dominated by implantable probes such as functionalised carbon nanotubes for H_2_O_2_ [8]. These devices rely on the chemical interaction of these nanomaterials with the desired biomarker, which leads to a change in the infrared spectrum. However, this technology requires the implantation of the nanomaterials and bulky and expensive equipment for the measurement of changes in infrared absorption, which limits its use in field applications.

New advances in the field of plant wearables have enabled measurements of physical parameters such as the flow rate in xylem, using microheaters [9], and the growth rate, through fibre optic sensors [10]. The use of nanomaterials includes carbon nanotubes decorated with silver nanoparticles [11], or plasmonic nanoparticles for the determination of leaf volatile fingerprints [12] have been adapted for the early detection of microbial diseases due to *Phytophthora infestans* or *A. solani*. These systems were assembled in the form of a pocket-sized device that could be used in combination with smartphones, and the authors measured a significant response within 1–2 days of inoculation with the pathogen. The determination of changes in leaf physiology and microclimate parameters have additionally been explored using electrodes printed onto flexible substrates [13,14,15]. Zhao et al. [13] developed a miniaturised (30 µm thick) and lightweight leaf sensor that could be used for the determination of leaf turgor and microclimate conditions including humidity, temperature, and light intensity. A primary goal in precision agriculture is the development of sensors capable of mapping spatiotemporal plant health at a high resolution. For instance, Yang et al. [16] developed an all-organic transparent plant e-skin for non-invasive phenotyping. A key advantage of this platform is its transparency, which solves a major issue from earlier work. However, this platform requires specialised microfabrication steps that limit scalability. Current advances in the field of wearable monitoring of leaf physiology involve the use of materials with low transparency and permeability, such as carbon electrodes, or require the implantation of conductive materials [17]. Recently, conductive polymers such as PEDOT:PSS have been extensively explored as flexible and stretchable electrodes for sensing applications due to the good biocompatibility [18] and conductivity [19]. They can be easily processed for the fabrication of thin electrodes through low-cost deposition methods such as drop-casting [20], spin coating [21], spraying [22], and screen printing [23], among others. This material has allowed the design of thin and transparent devices, for example, those used for the determination of ozone damage in *Vitis vinifera* L. plants [15]. Thus, the incorporation of conductive polymers for the fabrication of plant tattoo-based devices shows promise for the fabrication of low-cost and non-invasive detection of plant abiotic stress.

This trend toward less invasive devices is also seen in impedance-based hydration sensors. For example, electrical impedance apectroscopy has been applied to monitor indoor lettuce farming for 16 h periods [24]. However, the method relies on a complex Double-Shell Model for data interpretation, which is not a simple, direct readout of water status. To address this gap, foundational work continues on the electrical modelling and fabrication of transferable PEDOT:PSS-based capacitive sensors [25], which is essential for creating reliable, scalable, and more biocompatible devices that overcome the opacity and rigidity of older methods.

Other works have attempted to incorporate microclimate compensation to separate environmental noise from physiological signals. For example, flexible in vivo sensors have been developed to monitor the effects of a Vapour Pressure Deficit on water productivity [26]. This approach has evolved toward integrated systems, such as the development of a “hybrid multifunctional physicochemical sensor suite” designed for continuous crop health monitoring [27]. However, plant microclimates are highly influenced by external environmental conditions [28], hindering their applicability.

The leakage of ions such as K^+^ has been reported as a response to abiotic stresses including salinity [29], drought [30], and temperature sensitivity [31]. Such an electrolyte leakage is detectable almost instantaneously [32] and can be measured due to the changes in the conductance of the plasma membrane. As such, changes in the concentration of ions within the plasma membrane and the conductance can be indicative of the stress response of the plant. In tomato plants, a 2% increase in conductance of plasma membranes in leaves due to potassium leaking has been found as a consequence of water stress experienced for 20 days [33]. The determination of the electrical resistance of plant leaves therefore represents a powerful tool for the early detection of abiotic stresses such as drought. However, current methods for the determination of electrical conductance in leaves are invasive and require extensive treatment of plants [34].

To address the need for minimally invasive in situ monitoring, soft ionic–electronic interfaces have been developed, including biomimetic Organic Electrochemical Transistors (OECTs), which directly interface with plant tissues to determine variations in the ion content of the plant’s sap [35]. However, these sensors lacked the molecular specificity required for a precise diagnosis. Recent advancements have focused on refining this OECT technology for highly specific targets. This includes biocompatible sensors designed for the real-time measurement of the concentration and saturation of ions in plant sap [36]. A significant enhancement in specificity was achieved with implantable sensors capable of monitoring diurnal (day/night) cycles of xylem sap glucose and sucrose [37]. This allowed, for the first time, in vivo tracking of key metabolites. However, given the implantable nature of the sensor, wounding the plant tissue was still required. This elicited a defence that could limit long-term signal stability. More recently, this technology has been deployed to detect highly complex, low-concentration analytes with the development of sensors for the long term, continuous in vivo monitoring of phytohormones [38]. This work targeted specific indole- and phenol-derived hormones, but faces the challenge of maintaining high selectivity against a complex chemical background and avoiding sensor drift over extended periods. The practical application of these sensors is now being demonstrated, moving from component development to solving agricultural problems. Initial applications focused on the early detection of drought stress in tomato [39], successfully identifying stress signatures before visible symptoms appeared. This concept has been moved into the field to test its use for optimising crop water management [28]. However, challenges in scalability, long-term stability and energy consumption of devices remain.

The present work describes a miniaturised sensor that can simultaneously measure leaf ions and water content. We hypothesise that ultrathin, transparent PEDOT:PSS/PDMS hybrid films directly deposited onto living leaves can function as both sensitive impedance-based sensors, for ionic strength and the water content, and triboelectric nanogenerators, to self-power such systems, given the multifunctional nature of such materials. We further hypothesise that aerosol-based deposition will produce more homogeneous, conformal films than conventional drop-casting, resulting in improved sensor stability and signal reproducibility. As such, the final device incorporates a transparent and flexible PEDOT:PSS thin film deposited onto a plant leaf using a low-cost aerosol-based method. The low thickness of this conductive film led to a high flexibility and transparency. In addition, the sensor was protected using PDMS, improving the stability of the film against physical parameters such as high humidity. This film could also be used to generate a voltage through triboelectricity, leading to a high power generation due to the high surface area of leaves. We quantitatively validate these hypotheses by correlating electrical impedance with leaf water and the ion content under controlled environmental conditions using a low-cost device. This technology could enable the future design of self-powered sensors that can monitor ionic strength and water needs in plants. The final device could be used for the study of day–night changes in transpiration, and to predict water and fertiliser needs over time in tomatoes. There would be a plethora of applications in smart farming and the early diagnosis of plant diseases.

## 2. Materials and Methods

### 2.1. Materials

PEDOT:PSS and sodium chloride were purchased from Sigma-Aldrich (St. Louis, MO, USA). PDMS (Sylgard 184) was purchased from Dow Inc. (Midland, MI, USA) Liquid fertiliser was purchased from Hygeia Chemicals Ltd. (Oranmore, Ireland). Impedance analyser ad5933 was purchased from Analog Devices (Wilmington, MA, USA). A Wio Terminal microcontroller was purchased from SeeedStudio (Shenzhen, China). Silicone sealant was purchased from RS Components (Corby, UK). Kontakt Chemie Black Graphite Aerosol Conductive Lacquer was purchased from Kontakt Chemie (Iffezheim, Germany). An LM324 Operational amplifier was purchased from Texas Instruments (Dallas, TX, USA). An air pump was purchased from Pssopp (Shenzhen, China). An ultrasonic atomiser was purchased from Geekcreit (Shenzhen, China). Compost was purchased from Mokuzai (London, UK). Finally, tomato seeds (gardeners’ delight variety) were purchased from Thompson and Morgan (Ipswich, UK). Carbon-based electrodes for visualisation were deposited using a commercial solution (GRAPHIT 33), purchased from CRC Industries Deutschland GmbH (Iffezheim, Germany).

### 2.2. Fabrication of Printed Sensors onto Tomato Leaves

**Figure A1:**
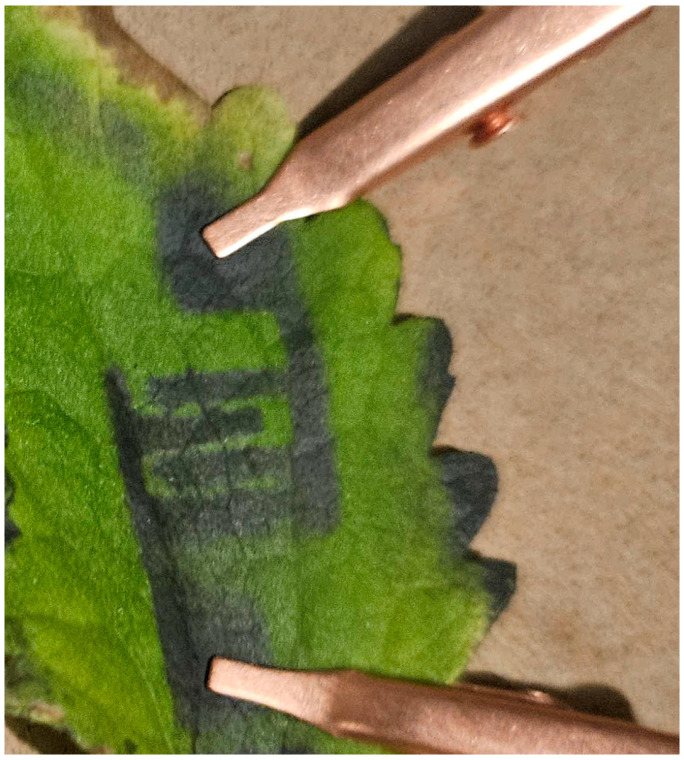
Connection of Wio Terminal device to leaf-deposited electrodes. Toothless (flat-jaw) crocodile clips were used to prevent mechanical injury to the leaf tissue.

The leaf-based sensors developed within the present work consisted of a thin PEDOT:PSS film deposited onto tomato leaves using an interdigitated electrode configuration. PEDOT:PSS films were deposited onto the tomato leaves using an aerosol deposition method. In this case, an air pump was connected to a reservoir with an incorporated ultrasonic sonicator. This reservoir contained the precursor solution: 10 mL of a solution of 1 wt% conductive PEDOT:PSS dispersed in ethanol. The generated aerosol was then directed towards the room-temperature substrate, generating a film. To allow a homogeneous fabrication of the conductive films, deposition cycles of 10 s were employed followed by 1 min of incubation with no deposition to enable the evaporation of the carrying solvent. In addition, a methacrylate-based mask was employed to pattern the electrodes directly onto the leaves. This mask was fabricated using laser cutting (PLS6.75, Universal Laser Systems, Scottsdale, AZ, USA), and it was designed as an interdigitated electrode, with 200 µm thick fingers and a distance between them of 1 mm, leading to a total surface area of 2 cm^2^. Electrical connection between the printed PEDOT:PSS interdigitated electrodes and the external circuit was established using toothless crocodile clips (flat-jaw type) to avoid mechanical damage to the leaf surface. For this purpose, the electrode mask design included two widened contact pads positioned at opposite ends of the interdigitated fingers. These pads provided a robust mechanical grip and ensured a stable, low-resistance electrical interface with the clips during impedance measurements. A photograph of this connection arrangement on an example electrode, coated with carbon to enhance visibility (compared to the transparent PEDOT:PSS thin film), is provided in Figure A1.

To improve the stability of the sensing devices, a protective PDMS film was additionally deposited onto the PEDOT:PSS electrodes. This protective film was deposited by the aerosol method reported here, by dissolving 100 mg of the elastomer in 10 mL ethyl acetate. This fabrication process was conducted for 60 s.

### 2.3. Characterisation of Printed Electrodes

After the deposition of the conductive PEDOT:PSS electrodes onto the tomato leaves, the physical properties of films were characterised. Initially, the thickness obtained by using this aerosol-based method was calibrated by depositing the PEDOT:PSS solution onto glass slides at different times. The glass slides had a mean roughness below 2 nm, and the thickness after deposition for 10, 30, 60, 120, 180, and 240 s was determined using a stylus profilometer (Dektakxt, Bruker, Coventry, UK). This calibration plot was used to estimate the deposited electrode thickness onto the tomato leaves. We used 25 cycles to deposit the final device onto plant leaves, leading to an electrode thickness in the range of 600 nm. The morphology of the films deposited onto the tomato leaves was studied by using scanning electron microscopy (SEM, EVO LS15, ZEISS, Oberkochen, Germany). The surface structure of the tomato leaves with no electrode films was first determined. The leaves were coated with a 50 nm thick gold film, and an accelerating voltage of 20 kV was used for the visualisation. The morphology of the leaves after deposition of the device containing the PEDOT:PSS electrode using the aerosol-based method was additionally determined and compared with the one obtained after drop-casting the 100 µL of the PEDOT:PSS dispersion.

The transparency of the aerosol-deposited electrodes at this thickness was determined to study the compatibility of the sensing device with the leaf function. In this case, the films were deposited onto a glass substrate, and the UV-Vis spectrum was measured between 400 and 100 nm (Lambda 750 S UV/VIS/NIR spectrometer, PerkinElmer, Waltham, MA, USA). Pristine glass substrates where no PEDOT:PSS films had been deposited were used as blanks.

To characterise the surface wettability and interaction between the polymer films and the substrate, water contact angle measurements were performed. The analysis was conducted on PEDOT:PSS films coated with PDMS. To distinguish the effects of film morphology from the underlying substrate microtexture, these hybrid films were prepared by both aerosol deposition and drop-casting onto two different substrates: (1) flat glass slides and (2) the abaxial surface of tomato leaves. The apparent water contact angle was then measured using an optical tensiometer (Attension, Biolin Scientific, Gothenburg, Sweden).

The specific composition of the sensing materials employed within the present work, involving a conductive PEDOT:PSS film and PDMS as a protective coating, also allowed the generation of power through triboelectricity. This triboelectricity could be triggered through the friction of the electrodes deposited onto a leaf with a different leaf where no PEDOT:PSS had been deposited. To increase the conductivity of the non-modified leaf, a conductive carbon-based coating was applied onto the side that was not put into contact with the PEDOT:PSS electrode during the triboelectric generation. Both leaves were moved in a contact–separation motion against each other at a frequency of 1 Hz, and both the open-circuit voltage and short-circuit current were determined using an electrochemical station (Autolab PGSTAT128N, Metrohm, Runcorn, UK). The performance of the devices fabricated using the aerosol deposition method was compared with the power obtained between two pristine leaves. Finally, to demonstrate the superiority of the aerosol method, driven by the higher surface area and homogeneity, the performance of these electrodes was compared with the one obtained after drop-casting PEDOT:PSS and PDMS onto the leaves.

The triboelectric properties of the PEDOT:PSS/PDMS–leaf hybrid electrodes were analysed in terms of transferred charge, surface charge density, effective capacitance, and equivalent dielectric thickness. The short-circuit current ($I_{SC}$), and open-circuit voltage ($V_{OC}$) were recorded under a periodic contact–separation frequency of 1 Hz. The charge transferred per cycle (*Q*) was obtained from the current–time data according to Equation (1) [40,41]:

(1) $$Q=\;\int I_{SC}\left(t\right)dt$$

The effective areal charge density was then calculated following Equation (2):

(2) $$\sigma_{eff}=\frac{Q}{A_{geo}}$$

where $A_{geo}$ represents the geometric electrode area (2 cm^2^). The effective capacitance of the device was estimated from that measured charge and voltage by Equation (3) [40,41]:

(3) $$C_{eff}=\frac{Q}{V_{oc}}$$

and an equivalent dielectric gap thickness was approximated from the classical capacitor relation provided in Equation (4):

(4) $$d_{eff}=\frac{\varepsilon_{0}A_{geo}}{C_{eff}}$$

where $\varepsilon_{0}$ is the permittivity of free space.

While the electrochemical station provided initial baseline characterisation, a portable and field-deployable measurement system was required for the mechanical resilience tests, which simulate long-term environmental exposure. This portable system was built using the Wio Terminal microcontroller (Seeed Studio, Shenzhen, China), leveraging its built-in Analog-to-Digital Converters (ADCs). The system was designed to measure both the open-circuit voltage (*V_OC_*) and short-circuit current (*I_SC_*) by switching between two distinct circuit modules 1. For open-circuit voltage measurement, the TENG output is characterised by high voltage and high internal impedance. To measure this without loading the circuit, a high-impedance voltage divider was used, constructed from one one-hundred-megaohm resistor and one one-megaohm resistor. This circuit provides a very high input impedance to approximate an open-circuit condition, while safely scaling the high voltage output, such as ten volts, down to the 0–3.3-volt readable range of the microcontroller’s analogue-to-digital converter. The raw value was then programmatically recalculated to the full open-circuit voltage based on the known divider ratio.

For short-circuit current measurement, the nano-ampere to micro-ampere current from the TENG was measured using a transimpedance amplifier circuit, built with an LM324 operational amplifier. The input of this amplifier, connected to the TENG, creates a virtual ground, which establishes a short-circuit condition. The transimpedance amplifier effectively converts the small input current into a measurable voltage via a one-megaohm feedback resistor, allowing the microcontroller’s analogue-to-digital converter to accurately record the current waveform.

### 2.4. Assembly of Sensing Device and Data Analysis of Impedance

**Figure A2:**
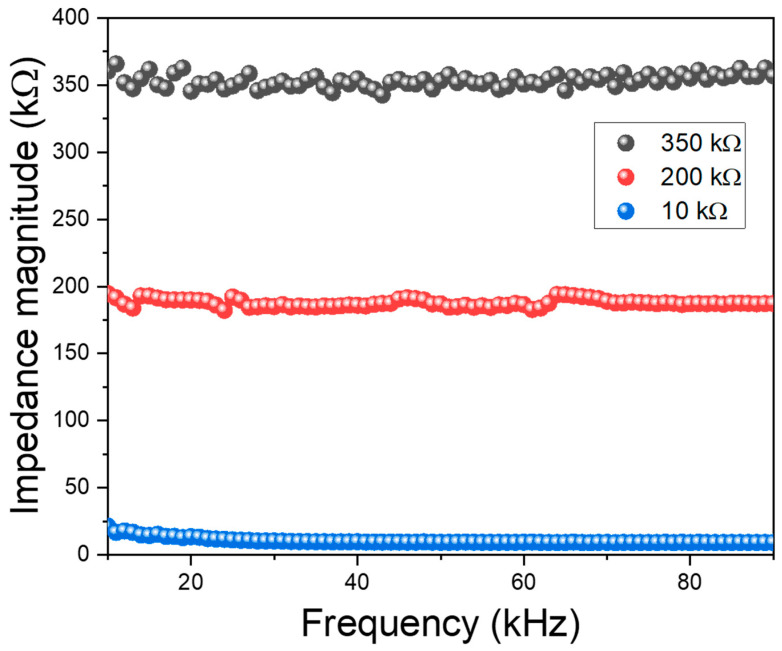
Recorded impedance magnitude within the frequency range of 10–90 kHz when using known resistors. The magnitude of the employed resistors was selected to demonstrate the accuracy of the device in the determination of impedance within the range of leaves, as is shown in the plot.

**Figure A3:**
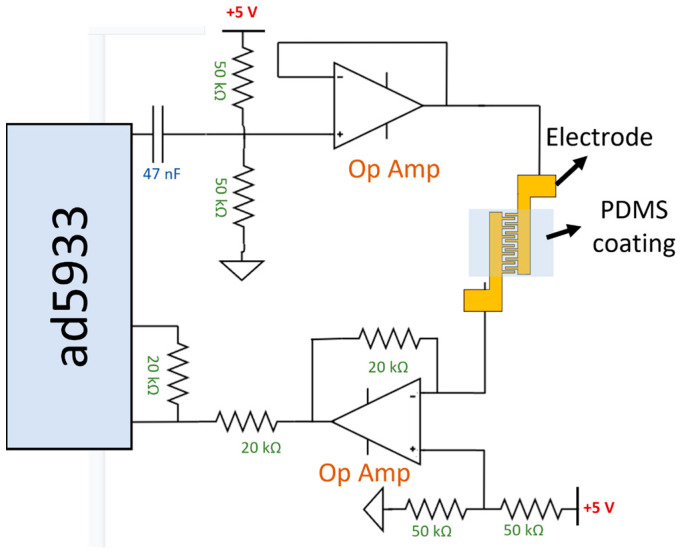
Schematical representation of the impedance measurement circuit employed in the present work. All resistances, capacitors, and operational amplifiers were used according to the manufacturer indications for bioimpedance measurements. The circuits were connected to the PEDOT:PSS electrodes for impedance measurements.

**Figure A4:**
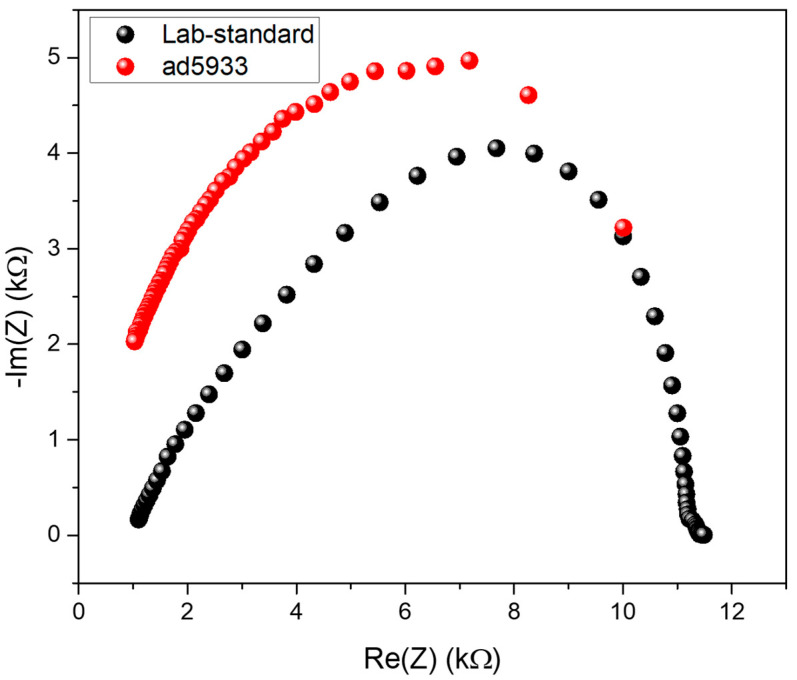
Comparison of results between low-cost ad5933 device and AUTOLAB lab-standard equipment.

The impedance of leaves was analysed by using an ad5933 impedance analyser (Analog Devices, Wilmington, MA, USA). The employed circuit was assembled according to the manufacturer’s guidance, as specified in Figure A2. This device was connected to a Wio Terminal for the data collection. In addition, Mueller toothless crocodile clips were used to connect the device with the PEDOT:PSS conductive film deposited onto the plant leaf. Initially, the device was calibrated using a range of known resistors (Figure A3). Benchmarking of impedance measurements was also conducted by comparing impedance results from those obtained by a lab-standard device (Autolab PGSTAT128N, Metrohm, Runcorn, UK), showing similar results (Figure A4). The device could determine the impedance of the reference materials within the desired range. This impedance was recorded within the frequency range of 10–90 kHz. The calibration was used to calculate the real and imaginary impedance values of the PEDOT:PSS electrodes, along with the changes due to the concentration of electrolytes. Both the capacitance and resistance could be estimated using the EIS analyser software v.1.0 reported by Bondarenko et al. [42]. An equivalent circuit consisting of a resistor and a capacitor in parallel was employed, and the simulation was performed with the LevMarq algorithm.

### 2.5. Calibration of Sensing Device in Plants

The sensors developed within this work could be used for the monitoring of both the ionic strength and the dryness of leaves through the impedance measurements. For the initial calibration of the devices, the leaf impedance values were recorded using the device developed within previous sections. The PEDOT:PSS and PDMS films were deposited onto a 6-week-old tomato leaf, and a peristaltic pump was connected to the stem of a cut, aerial section of a plant. This peristaltic pump was used to determine the changes in leaf impedance due to the concentration of ions. In this case, different solutions containing known concentrations of NaCl ranging from 0.1 mM up to 0.1 M were pumped. The impedance of the plants was then recorded using the Arduino-based device every 20 min.

The ability of this device to determine changes in leaf dryness was additionally studied. In this case, DI water was pumped through the plant stem using the peristaltic pump. After 1 h of continuous pumping, the pump was removed from the stem, and the leaves were weighted.

Relative leaf weight (%) was determined gravimetrically to evaluate relative water loss as an indicator of transpiration and dehydration. Mature tomato leaves were carefully detached from the plants and immediately weighed using a precision analytical balance. Leaves were placed on a fine mesh, and their weight was recorded at fixed 60 min intervals. The percentage weight loss (ΔW%) was calculated as Equation (5).

(5) $$∆W\%=\left[\frac{W_{o}-W_{t}}{W_{o}}\right]\times 100$$

where *W_O_* is the initial fresh weight, and *W_t_* is the weight at time *t*. This gravimetric approach is a standard and physiologically relevant method for assessing passive water loss through stomatal and cuticular transpiration. Similar detached-leaf assays have been successfully applied in several plant studies [43,44,45].

Finally, the effect of humidity on the performance of the sensors was studied. In this case, the tomato plant was enclosed in a sealed box, where a bme680 environmental sensor had been placed. The humidity of the chamber was then gradually increased by using a water aerosol generated as described within the previous sections. The impedance of the leaf was then measured at each humidity value.

Environmental parameters were continuously monitored during the diurnal experiments to ensure stable measurement conditions. The temperature, relative humidity, barometric pressure, and volatile organic compound (VOC) concentration were recorded using a BME680 environmental sensor (Bosch Sensortec, Reutlingen, Germany) integrated with a Wio Terminal microcontroller, which also provided a built-in light intensity sensor. Soil moisture was measured using a capacitive humidity probe that was calibrated with soil samples of a known gravimetric water content to determine the relative field capacity. All parameters were logged at one-minute intervals throughout the measurement period. During the 24 h recordings, temperature ranged from 25.5 to 28.0 °C, and relative humidity ranged from 60.3% to 66.5%.

### 2.6. In Vivo Testing of Electrodes Directly Fabricated onto Leaves

To demonstrate the applicability of the sensing devices in the monitoring of plants in vivo, the PEDOT:PSS electrodes were deposited onto 6-week-old tomato leaves. The tomatoes were kept in peat-free compost, and they were watered every 48 h. The design of our sensing device allowed the monitoring of the leaf status within the short and long terms, for at least 9 days from deposition. To test the ability to monitor the films within the short term, the impedance of leaves was measured every 20 min. Initially, the changes in ionic strength and relative leaf weight changes due to the watering were recorded. The base concentration of electrolyte in the plant leaf was first determined for 2 h. The plants were then watered with 100 mL of a concentrated solution containing 0.1 M NaCl in DI water, and the changes in ionic strength were recorded for 3 h. Finally, 100 mL of DI water was used, and the impedance was additionally measured every 20 min for 3 h. The daily variations in both ionic strength and relative leaf weight due to the natural day–night cycles of the plant were separately determined. In this case, the leaf impedance was recorded. The tomato plant used within these experiments had not been watered for 24 h prior to the time where the impedance measurements were taken.

The device described in this work also enabled long-term monitoring of leaf physiology. For this study, PEDOT:PSS/PDMS electrodes were fabricated onto two separate tomato plants. Both plants were irrigated with DI water every 48 h throughout the monitoring period. Impedance measurements were recorded every 20 min over a 2 h window each day during stable daytime greenhouse conditions. Following the initial period of DI-water irrigation, one plant received a single fertiliser supplementation on Day 6 (5 mL of DI water containing 10 µL of liquid fertiliser; composition provided in Table A1), while the second plant continued to receive only DI water. Monitoring was continued for a total duration of nine days.

## 3. Results and Discussion

### 3.1. Leaf Sensor Fabrication

The method of deposition of electrodes onto plant leaves is a crucial step in the production of devices as the surface morphology and thickness influence its final performance: the quality of adhesion and surface area are important factors. The electrodes designed in this work were made of a PEDOT:PSS conductive film coated by PDMS, exploiting properties of this material including hydrophobicity (enhancing adhesion to leaf surface) [46], elasticity [47], gas permeability [48], and transparency when used in the form of thin films [16,49]. This material could improve the electrical properties of the leaf whilst ensuring a proper permeability, which is crucial for the physiological activity.

**Figure 1:**
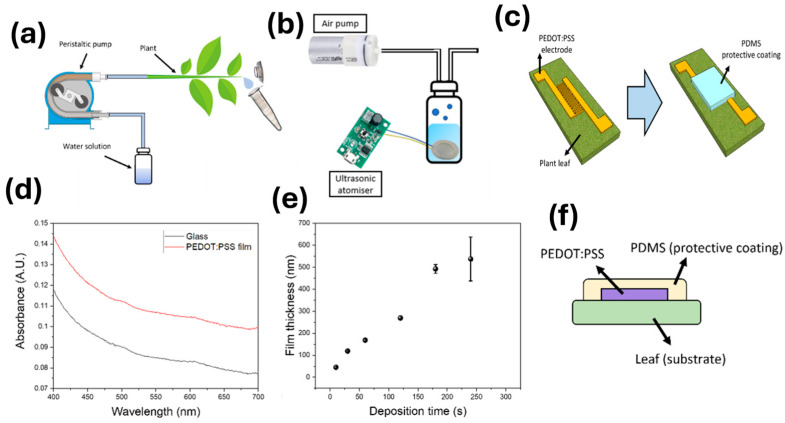
(**a**). Setup for the calibration of the final devices deposited onto the plant leaves. The use of the peristaltic pump allowed the control of the concentration of electrolytes inside the plant stems, which could be used for the calibration of the device. (**b**). Aerosol deposition setup used for the fabrication of the sensing films developed within this work. An ultrasonic atomiser was employed for the generation of the aerosol, which could be directed towards the substrate by employing an air pump. (**c**). Schematical representation of the device structure, containing the interdigitated electrodes for the sensing. After fabrication of the flexible and transparent electrode, the device was coated using a PDMS elastomer to ensure the stability of the device (**d**). Absorbance of the films characterised by UV-Vis spectroscopy within 400–700 nm range, demonstrating the transparency of the sensing films. (**e**). Thickness calibration of the deposition setup, comparing different deposition times. (**f**). Structure of the final device, where the PEDOT:PSS conductive film has been fabricated by aerosol deposition directly onto the leaves. A PDMS-based protective coating is then placed to improve the stability of the films.

To obtain a good control over the film dimensions, and to allow the fabrication of patterned electrodes, an aerosol-based deposition was used. The aerosol deposition setup consisted of a water reservoir containing an ultrasonic sonicator in a solution of 1 wt% conductive PEDOT:PSS, along with an air pump. The ultrasonic sonicator generated an aerosol that could be directed towards the tomato leaf (Figure 1a). In this case, an interdigitated electrode configuration was devised, using a methacrylate-based mask. A thin PDMS film was additionally deposited as a protective coating against environmental factors (Figure 1b). The testing of the miniaturised printed sensors was carried out using a custom setup. A peristaltic pump was connected to a plant stem, and a solution with a known concentration of electrolytes was pumped through the plant stem (Figure 1c). This system allowed the calibration of the electrodes in terms of changes in impedance due to increases in electrolyte concentrations.

Initially, the deposition conditions of the PEDOT:PSS-based conductive films were optimised. In this case, the PEDOT:PSS was deposited onto a glass substrate, and the thickness of the films fabricated at different times was determined using a stylus profilometer. The thickness of the films increased at an approximate rate of 2.6 nm/s (Figure 1d). As such, this low-cost method could be used for the deposition of nanometrically thin films with controlled dimensions. To ensure a proper conductivity of the electrodes, while offering a good flexibility, miniaturised sensors with a thickness of 600 nm were developed. In addition, these sensors showed a good transparency with a maximum absorbance of 0.026 a.u. at 400 nm, obtained by measuring the UV-Vis spectrum of a PEDOT:PSS film deposited onto a glass substrate (Figure 1e). The low absorbance minimised disruption to photosynthesis taking place in the plant leaves.

The final sensing device consisted of thin PEDOT:PSS films deposited onto the plant leaves with a PDMS coating (Figure 1f). This coating was also applied using the reported aerosol method. Similar to the case of the PEDOT:PSS electrodes, a concentration of 1 wt% of PDMS was diluted in ethyl acetate. The use of PDMS as a protective coating prevented interference from environmental humidity, whilst improving the PEDOT:PSS robustness. In addition, PDMS is a gas-permeable material, which allows the natural gas exchange of the leaves [48].

**Figure A5:**
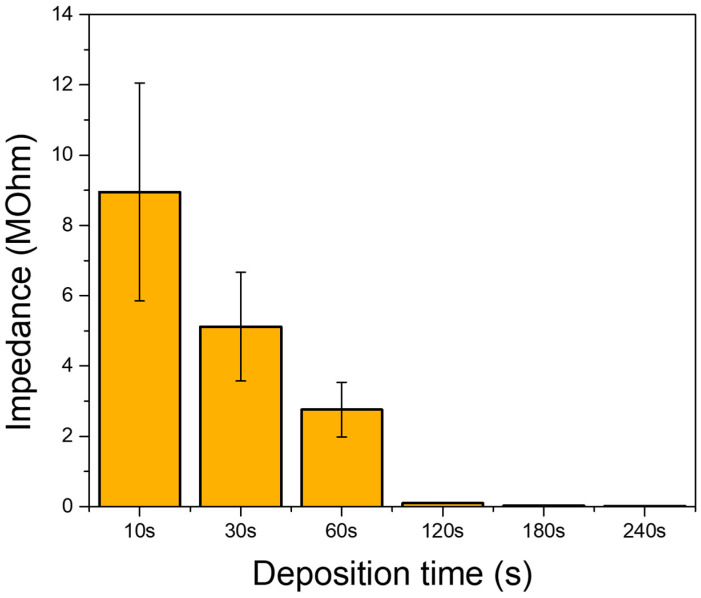
Relationship between PEDOT:PSS film impedance and deposition time. The plot shows a non-linear decrease in impedance as film thickness increases, corresponding to the formation of a conductive percolation network.

To evaluate the effect of electrode thickness on electrical performance, the impedance of PEDOT:PSS films deposited at different times was measured using the AD5933 analyser (Figure A5). At deposition times below 60 s (≈200 nm), the films exhibited very high impedance values (>1 MΩ), which suggested a low thickness, along with an incomplete coverage and insufficient percolation of the conductive network, as reported in previous work [50]. With increasing thickness, impedance decreased markedly, stabilising between 10 kΩ and 100 kΩ at approximately 600 nm. This range aligns with the optimal sensitivity and measurable window of the AD5933 module, allowing accurate impedance tracking while avoiding signal saturation. Therefore, the selected 600 nm thickness offers an effective compromise between electrical performance and biological compatibility, leading to a good optical transmittance and decreasing the invasiveness of the sensor for the plant.

### 3.2. Characterisation of the Miniaturised Sensors

**Figure 2:**
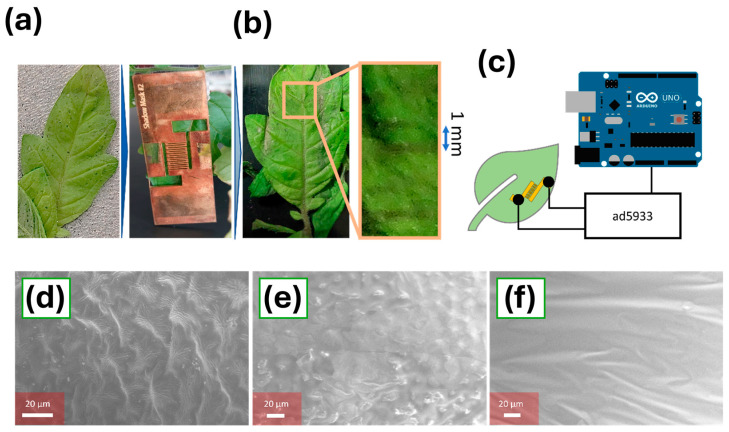
(**a**). Representation of the deposition workflow. A picture of the pristine leaf before deposition is shown. A methacrylate-based mask was used for the deposition of PEDOT:PSS conductive electrodes. Final structure of modified leaf containing the printed electrodes. (**b**). A detailed picture of the electrode interdigitated structure with 1 mm separated fingers is shown. (**c**). Schematical representation of the microcontroller-based device for the determination of plant impedance. A low-cost ad5933 impedance meter was employed in contact with the conductive electrode. The interdigitated electrodes incorporated two enlarged terminal pads on each side to enable gentle electrical connection using flat, toothless crocodile clips. (**d**). Scanning electron microscopy imaging of the surface of a tomato leaf before the deposition of the conductive electrode. (**e**). SEM imaging of the surface of the tomato leaf after the deposition of the PEDOT:PSS thin film. (**f**). SEM imaging of the surface of a tomato leaf after the deposition of the PEDOT:PSS thin film using drop-casting as the fabrication method.

For the final fabrication of the sensing electrodes onto the plant leaves, a custom-made deposition mask was designed. This mask was developed by patterning a methacrylate film by laser cutting, and it could be placed onto pristine plant leaves for the deposition (Figure 2a). The mask consisted of seven interdigitated 200 µm wide fingers, separated by 1 mm (Figure 2b). The PDMS coating was then applied to the whole device. A microcontroller-based device, based on the ad5933 impedance meter, was used for the determination of the leaf ionic strength and dryness (Figure 2c). This device allowed the study of the changes in the leaf conductivity, which could be correlated with the ion concentration and the water content of leaves following previous work reported in the field [51]. A schematical representation of the circuit employed for the impedance measurements is provided in Figure A2.

The morphology of the deposited films onto the leaf surface was studied using scanning electrode microscopy. Before the deposition, the leaf presented a rough surface (Figure 2d and Figure A7). This surface roughness could be preserved by the deposition of a thin PEDOT:PSS film using the aerosol method, which increased the effective area of the sensor (Figure 2e). Tomato leaves present a highly microstructured surface covered with dense trichomes and irregular epidermal ridges. Although visualising these trichomes by SEM is challenging, since gold sputtering and vacuum exposure can collapse or damage these fragile structures, the morphology observed in SEM images after coating them with the PEDOT:PSS/PDMS films provided clear evidence of a uniform coating, showing ridges and epidermal features ranging between 0.5 and 1.2 µm. Additionally, the intersection between adjacent electrode fingers on the leaf was visualised (Figure A7). Aerosol-deposited electrodes display characteristic “mountains and valleys” with peak-to-valley distances of approximately 19–25 µm, consistent with trichomes and epidermal protrusions encapsulated beneath the conductive film. This observation demonstrates that the aerosol process enables conformal coverage of rough and trichome-rich plant tissues, rather than forming isolated clusters or delaminated regions. These results are consistent with our previous work on aerosol-deposited polymer electrodes for complex or flexible substrates, which demonstrated excellent film uniformity and mechanical integrity on different electrode geometries [52,53]. To further assess film homogeneity, a comparative analysis between aerosol-deposited and drop-cast films was performed. Aerosol-deposited films exhibited a more uniform morphology and lower surface roughness (average roughness: 23 nm, Figure A8a), preserving the natural microtexture of the leaf. In contrast, drop-cast coatings showed severe thickness gradients (average roughness 198 nm, Figure A8b) when studied using a stylus profilometer. These morphological differences explain the higher reproducibility and stability of the aerosol-fabricated electrodes during impedance and triboelectric measurements.

Finally, to characterise the interaction between the PEDOT:PSS/PDMS films and the leaf substrate, additional wettability analyses were performed. Pristine tomato leaves exhibit a mean roughness of 24.73 µm and a water contact angle of 97.67°, as studied by Ma et al. [54]. On the contrary, in our work, aerosol-deposited PEDOT:PSS films exhibited an initial contact angle of 41.6 ± 3°, reflecting the intrinsic hydrophilicity of the polymer. However, when PDMS was deposited onto the PEDOT:PSS layer, the resulting hybrid films displayed a contact angle of approximately 90° on PDMS prior to leaf deposition. The contact angle was similar on films deposited by aerosol and drop-casting on a flat substrate (aerosol: 91.7 ± 2°; drop-cast: 90.7 ± 2°). However, following deposition onto tomato leaves, the apparent contact angle increased to 105.7 ± 1° on aerosol-deposited films, while it stayed at a value of 91.4° ± 1° when PDMS was deposited by drop-casting (Figure A9a,b). This divergence in wettability behaviour indicates that the aerosol-deposited films conform to the underlying leaf microstructure, whereas the thicker and less uniform drop-cast films do not achieve such conformal coverage.

The increase in surface area due to conformational coating not only improved the final sensitivity but also increased the electrical output of the device when operated as a triboelectric generator. The importance of using this aerosol-based method was demonstrated further by imaging the surface morphology of an electrode that had been deposited using a drop-casting technique. In this case, 10 µL of the same PEDOT:PSS concentration used during the aerosol-based fabrication was deposited by drop-casting and dried overnight. A thickness of 3.7 ± 0.1 µm with a significantly lower surface area was obtained (Figure 2f). Due to the higher surface area, and the possibility of controlling the film thickness with higher precision, the low-cost aerosol-based deposition method was chosen for the fabrication of the devices.

**Figure A6:**
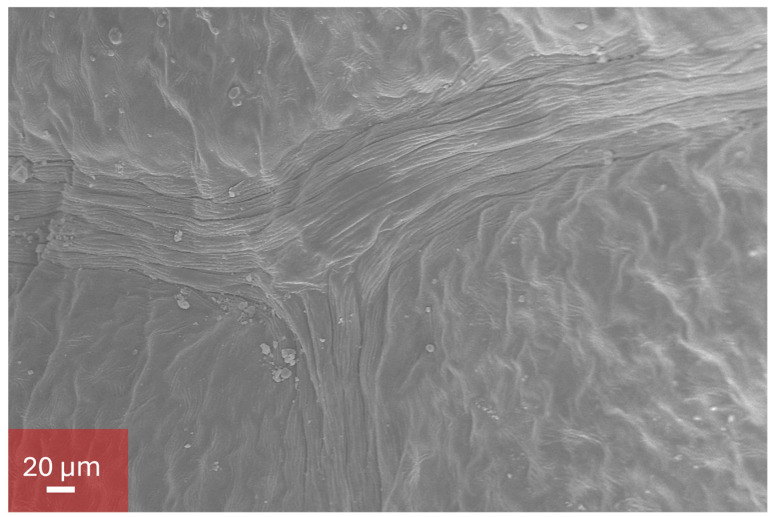
Scanning electron micrograph (SEM) of the pristine, uncoated tomato leaf surface. The image shows the inherent rough microtopography used as a baseline before the aerosol deposition of the PEDOT:PSS/PDMS film.

**Figure A7:**
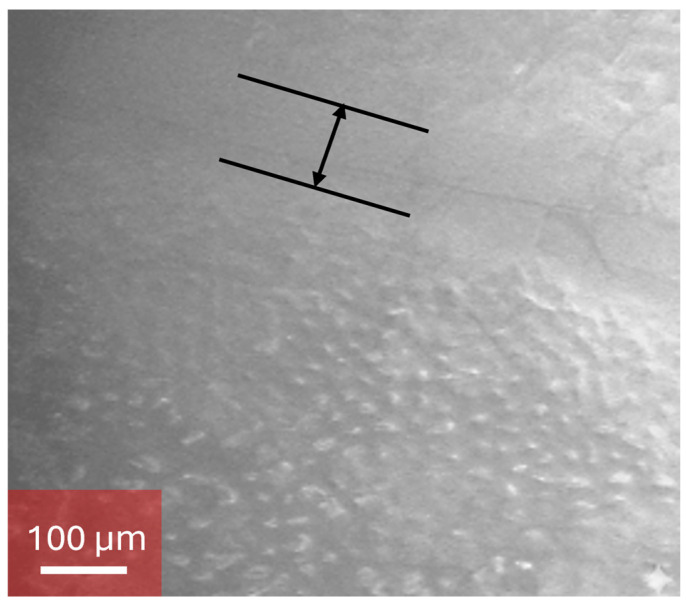
Scanning electron micrograph (SEM) of sensor-coated tomato leaf surface. Arrows indicate the space between electrode fingers.

**Figure A8:**
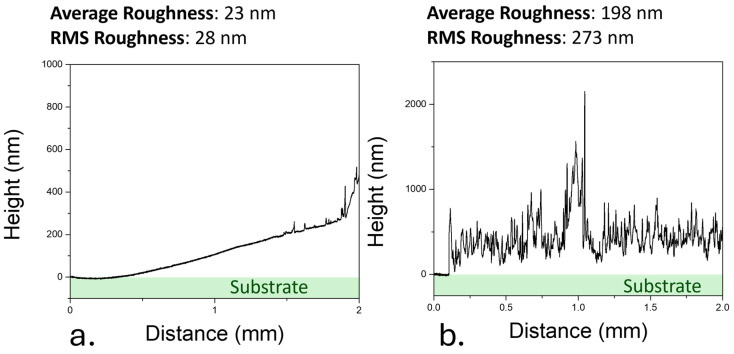
(**a**). Profilometer analysis of an aerosol-deposited PEDOT:PSS film, showing a uniform morphology, with low average roughness (23 nm). (**b**). Profilometer analysis of a drop-cast PEDOT:PSS film, revealing severe thickness gradients and a high average roughness (198 nm).

**Figure A9:**
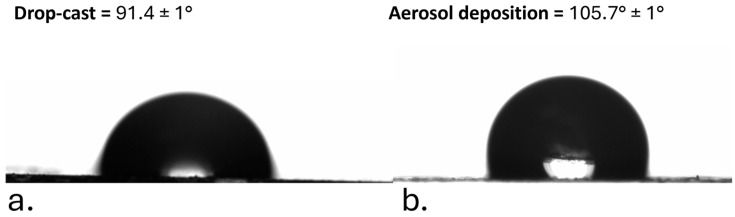
(**a**). Water contact angle of a drop-cast PDMS film on a tomato leaf. The angle (91.4 ± 1°) remains similar to that of a flat substrate, indicating a non-conformal coating that masks the leaf’s natural microtexture. (**b**). Water contact angle of an aerosol-deposited PDMS film on a tomato leaf. The apparent contact angle increases to 105.7 ± 1°, showing that the film has conformally coated the leaf’s underlying microstructure.

**Figure A10:**
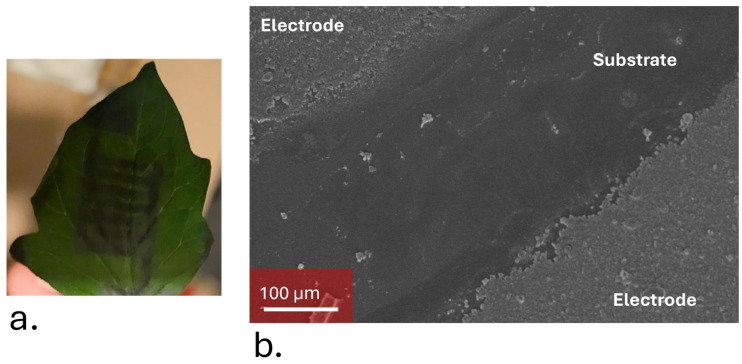
(**a**). Optical image of the interdigitated carbon-based electrode, showing the high precision and uniformity of the aerosol-deposited pattern. (**b**). SEM image of the interdigitated carbon-based electrode, confirming the fine pattern definition and consistent film thickness.

The high optical transparency of the aerosol-deposited PEDOT:PSS films (absorbance 0.145 a.u. at 400 nm) makes direct optical or SEM visualisation of patterned electrodes particularly difficult, since ultrathin films provide little contrast against the leaf surface. To demonstrate the fidelity of the aerosol-based patterning method, an additional electrode was fabricated using an optically opaque carbon-based conductive paint under identical aerosol and mask conditions. The resulting interdigitated pattern can be clearly observed in the optical image presented in Figure A10a, which confirms the high precision and uniformity of the deposited fingers. SEM imaging of the same carbon-based electrode further verified the fine pattern definition and consistent film thickness across the electrode area (Figure A10b), similar to the pattern obtained previously when PEDOT:PSS was deposited onto plant leaves (Figure A6).

**Figure 3:**
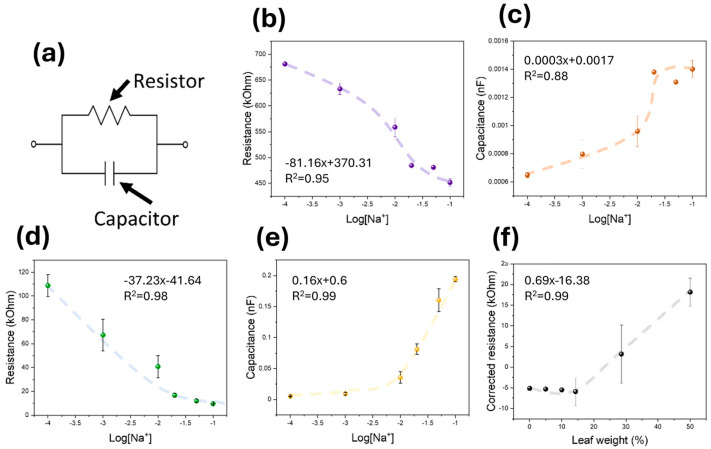
(**a**). Model circuit employed for the determination of the resistance and capacitance of the plant tissues. (**b**). Resistance values obtained after the calibration of the leaves without sensing devices using different concentrations of NaCl within the pumped fluid. (**c**). Capacitance values obtained within the leaves with no incorporated sensing devices. (**d**). Resistance values obtained in the leaves with a fabricated miniaturised device. (**e**). Capacitance values of the sensing devices subjected to different concentrations of NaCl. (**f**). Corrected resistance value of a drying leaf, calculated from that measured resistance and the calculated resistance using the capacitance. Calibration parameters are shown.

After the fabrication of the sensing electrodes, the devices were calibrated using different concentrations of NaCl within the stem. In this study, both the capacitance and resistance of the leaf tissue were calculated by fitting the obtained impedance spectrum using a single model of a resistor and capacitor in parallel (Figure 3a). This model has previously been employed for the characterisation of animal tissues [55], and it could be used for the simplified calculation of two parameters: leaf dryness and ionic strength.

When leaves where no PEDOT:PSS electrodes had been deposited were tested, the impedimetric response of the tissues changed upon increasing the NaCl concentration within the running fluid. This impedimetric change was reflected by the lower resistance and higher capacitance of the electrodes (Figure 3b,c). In this case, the electrical resistance decreased from 680 kOhm when 0.1 mM NaCl was used to up to 450 kOhm at 0.1 M. The electrical resistance of the leaf decreased from 108.8 kOhm down to 9.6 kOhm (Figure 3d), showing a higher sensitivity than in the case of pristine leaves. However, the capacitance of the films increased (Figure 3e), suggesting a change in the leaf behaviour from electrical resistor to a capacitor due to the higher concentration of ions. This behaviour was also observed in the case of the sensors incorporating the PEDOT:PSS films. In addition, the measured capacitance increased by almost two orders of magnitude. As such, the PEDOT:PSS thin film greatly improved the sensitivity of the films, whilst expanding the linear range of the capacitance increase up to 10^−4^ − 1.7 × 10^−2^ M. Beyond this concentration, the capacitance reached a plateau, consistent with saturation of available charge-transfer sites.

As observed, the ionic concentration of the plant stem triggered changes in both the resistance and the capacitance of the electrodes. However, when the weight of the plant decreased due to the loss of water alone, only the resistance of the leaf increased, with no significant change in plant capacitance. As such, this method could also be used to estimate the relative leaf weight (%) by correcting the resistance values using the corresponding capacitance for the calculation. In this case, we determined both the resistance and the capacitance employing the same circuit model, and the measured capacitance was used to determine the increase in resistance due to the water loss. We calculated the weight loss of the leaf by leaving the leaf unconnected to the pumping system under room-temperature conditions and measuring the changes in the weight. We then calculated the resistance of the leaf related to the ion concentration by using the calibration plot under a continuous flow of saline solution and subtracted this calculated resistance from that measured after drying the leaf. The resulting resistance could be used to track the relative leaf weight (Figure 3f).

At low relative leaf weight loss rates (<20%), the corrected resistance was negative. This was attributed to the high pressure within the plant stem that was obtained when the pump was used for the calibration of the sensors under saline conditions. This pressure increased the water content inside the leaves, further decreasing the resistance. However, this device could determine the relative leaf weight in the 15–50% range, which could be used to quantitatively evaluate the water needs of the plants. The capacitance derived from the Cole equivalent circuit represents more than a simple function of ionic strength in the surrounding electrolyte. In biological tissues, this term reflects the collective dielectric polarisation of the apoplastic and symplastic compartments, including contributions from cell membranes, cell walls, and intracellular ionic gradients. As ions accumulate or redistribute within and around cells, the polarisation at the cell–wall–membrane interfaces changes, resulting in measurable variations in apparent capacitance [56,57]. In this context, an increase in capacitance indicates enhanced ionic availability and membrane polarisation, typically associated with higher hydration or nutrient content, while a decrease corresponds to reduced ion mobility under water or nutrient stress. Therefore, the capacitance component obtained from Cole fitting provides an integrated indicator of the ionic and dielectric state of the leaf tissue, complementing the resistance term, which mainly reflects ionic conduction through the apoplastic fluid. This interpretation bridges the calibration performed with NaCl solutions and the complex in planta behaviour, reinforcing the physiological significance of the impedance spectra recorded by the PEDOT:PSS/PDMS leaf tattoo sensors.

As observed, the performance of the leaf tattoo sensor is governed by the morphology of the sensor–leaf biointerface. This is determined by the aerosol-based deposition method. Unlike conventional drop-casting, aerosol deposition produces a highly conformal, nanometrically thin (~600 nm) PEDOT:PSS film that closely replicates the complex topography of the tomato leaf surface, including epidermal ridges and trichomes. This conformality maximises the effective surface area of the electrode. The tomato leaf itself does not act as an electronic conductor; instead, its surface behaves as an ionic medium due to the continuous film of apoplastic water and dissolved electrolytes that occupies the extracellular spaces between epidermal and mesophyll cells [58,59]. These electrolytes form a hydrated network that supports ionic conduction along the leaf surface. When an AC potential is applied across the electrodes, charge transfer occurs through this thin hydrated apoplastic layer. Ionic oscillation and interfacial polarisation within this layer produce the impedance spectra measured by the sensor, consistent with earlier bioimpedance studies showing that plant tissues act as ionic conductors embedded in dielectric matrices [17,57,60].

**Figure A11:**
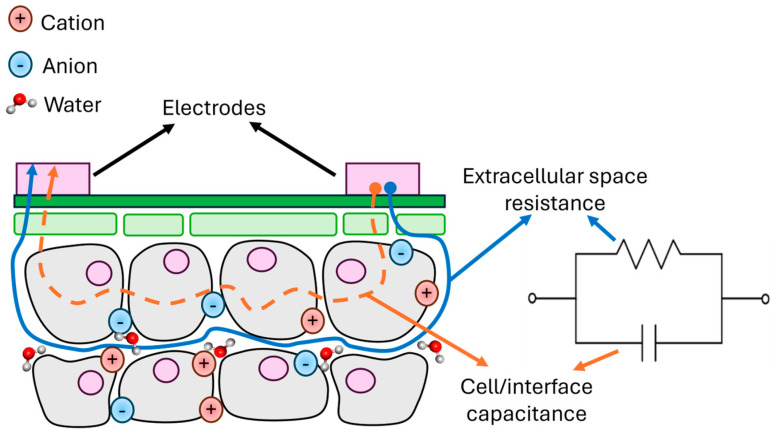
A schematic representation of the leaf tissue cross-section, illustrating the primary current pathways and the corresponding labelled equivalent circuit model used for impedance analysis.

To optimise sensitivity to this interfacial ionic transport, we adopted an interdigitated electrode (IDE) design. IDEs generate alternating electric fields that penetrate only a few micrometres into the surface, effectively probing the hydrated apoplastic region. The high density of finger pairs increases the active surface area and the fringe-field overlap, enhancing capacitive and resistive coupling with the ionic medium while keeping the device fully planar and non-invasive. Such architectures have been used in impedance-based biosensors for their high sensitivity to changes in local ion concentration and water content [61]. On the leaf, this geometry also facilitates conformal aerosol deposition and maintains pattern fidelity over ridges and trichomes, ensuring a continuous coverage and reproducible impedance readings. The resulting current pathway, illustrated schematically in Figure A11, involves ionic conduction through the apoplastic film between adjacent fingers and capacitive coupling across the cell wall interfaces. The effective impedance therefore depends on both ion mobility and local hydration, explaining the observed correlation between resistance, capacitance, and physiological parameters such as transpiration and nutrient availability. As such, for sensing (Mode 1), the enlarged electrode area enhances the capacitive and resistive coupling with the apoplastic environment. The conformal, high-area aerosol-deposited film facilitates uniform ionic coupling over the entire leaf surface, minimising local impedance artefacts. This stable interface enables the decoupling of resistance- and capacitance-dominated contributions, allowing us to independently track water loss dynamics and changes in ionic strength associated with nutrient availability. The system can be modelled as a parallel resistor–capacitor (R‖C) circuit, where capacitance correlates with ionic strength and resistance reflects the leaf water content. This modified Cole model enables the decomposition of impedance spectra into contributions from extracellular ionic conduction, cell membrane polarisation, and intracellular pathways [62,63,64]. Such a model was chosen to provide a physiologically meaningful framework, where the resistances map onto ion transport and hydration state variables, while the capacitive/CPE component captures dielectric and membrane-related elements. Moreover, the high surface area of the aerosol-deposited film ensures stable and sensitive impedimetric responses, enabling us to deconvolute these two physiological parameters. This capability allowed real-time monitoring of day–night transpiration cycles and early detection of nutrient depletion under deionised water irrigation.

For energy harvesting (Mode 2), the high surface area and conformal replication of microtextures also explain the enhanced power output (7.2 µW cm^−2^) observed in triboelectric mode. The rougher, microstructured aerosol-deposited films produced significantly higher power than smoother drop-cast electrodes (4.9 µW cm^−2^), confirming the direct role of morphology in triboelectric performance.

Film thickness further modulates both conductivity and optical transparency. Thinner coatings (<200 nm) showed discontinuous conduction paths and high impedance (>1 MΩ), while the 600 nm films offered a robust percolated network with impedance values in the 10–100 kΩ range, well-matched to the sensitivity window of the AD5933 analyser. This thickness also maintained high optical transparency while ensuring minimal interference with photosynthetic activity. Thus, 600 nm represents an optimal compromise between electrical performance, reproducibility, and biological compatibility.

**Figure A12:**
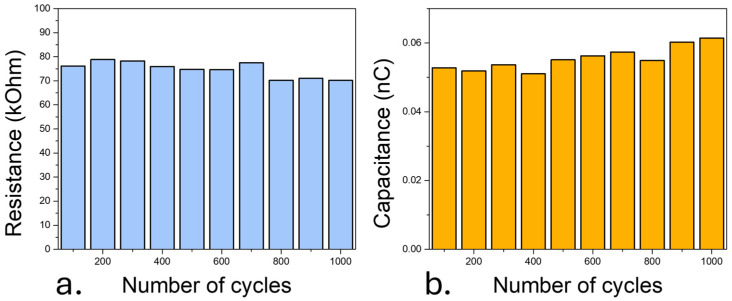
Impedance magnitude recorded after intervals of 100 rubbing cycles (up to 1000 cycles). The results show no measurable change in resistance (**a**) or capacitance (**b**), confirming that the electrical performance of the electrodes is unaffected by repeated mechanical perturbation.

Finally, to assess the stability of the sensor under mechanical perturbation, such as leaf movement caused by wind or handling, the PEDOT:PSS/PDMS-coated leaves were subjected to 1000 rubbing cycles at a frequency of 1 Hz. This motion mimicked the contact–separation dynamics used during triboelectric energy-harvesting measurements. The performance of both sensing (Mode 1) and energy harvesting (Mode 2) was evaluated. The impedance of the electrodes (Mode 1) was recorded after every 100 cycles using the AD5933 analyser. Results (Figure A12) showed no measurable change in impedance magnitude across the test duration, confirming that the electrical performance of the electrodes remains unaffected by repeated mechanical interaction.

**Figure A13:**
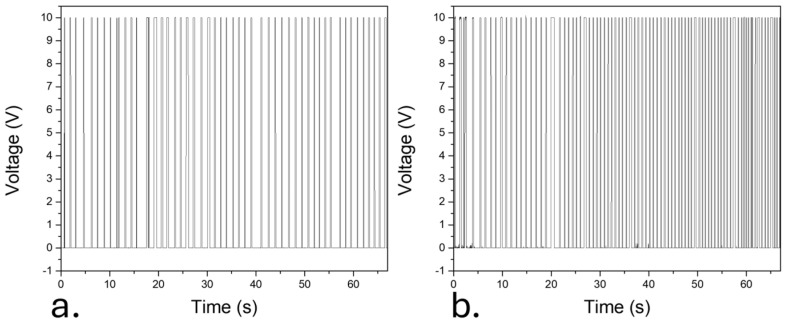
(**a**) Triboelectric voltage output (Voc) measured during the first-cycle mechanical test. (**b**) Triboelectric voltage output measured during the last-cycle mechanical tests and during the 1000-cycle mechanical test. The output remains highly stable, consistently producing a maximum voltage of approximately 10 V.

Similarly, the triboelectric power output (Mode 2) was measured throughout the cycling test and also remained highly stable, consistently producing a maximum open-circuit voltage of approximately 10 V, leading to a similar power density (Figure A13a,b). The PDMS overlayer contributes significantly to this dual-mode stability by providing elastic protection and maintaining intimate adhesion between the conductive PEDOT:PSS network and the microstructured leaf surface, consistent with previously reported work [65]. This mechanical resilience, together with the film conformal morphology, indicates that the sensor can operate reliably under the continuous movement conditions typical of outdoor or greenhouse environments.

### 3.3. In Vivo Testing of the Sensors

To determine the suitability of the sensors for the in vivo monitoring of plants, the sensors were deposited onto a 6-week-old tomato plant. These sensors could not only be operated for the determination of the plant ionic strength and dryness (first mode), but they could also be used to generate power through triboelectricity (second mode). This ability to produce electrical power was a consequence of the film composition, containing PEDOT and PDMS, and could be used in future designs to avoid the need for expensive batteries when conducting experiments in field applications.

**Figure 4:**
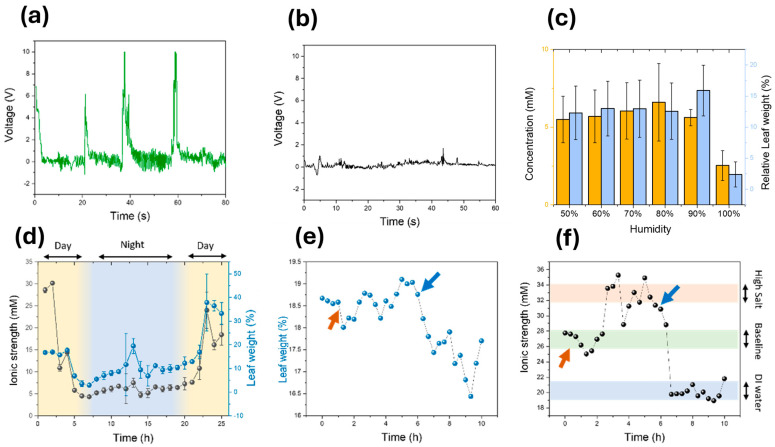
(**a**). Voltage output of the triboelectric generator developed by aerosol deposition of PEDOT:PSS onto a tomato leaf. (**b**). Voltage output generated between two leaves where no PEDOT:PSS electrodes or PDMS had been deposited. (**c**). Study of the interference of the devices from the environmental humidity. The plant ionic strength and relative leaf weight were estimated from the resistance and capacitance of the devices. (**d**). Continuous 24 h monitoring of the tomato plant, evidencing a change in both the ionic concentration and relative leaf weight during the daytime compared to night. (**e**). Estimation of the relative leaf weight under standard conditions, and after watering the plant with a highly saline solution and DI water. Red and blue arrows indicate the points where the plant was watered with 0.1 M NaCl and DI water, respectively. (**f**). Calculated ionic strength using the impedance data of the plant subjected to high-saline conditions and DI water. Red and blue arrows indicate the points where the plant was watered with 0.1 M NaCl and DI water, respectively.

**Figure A14:**
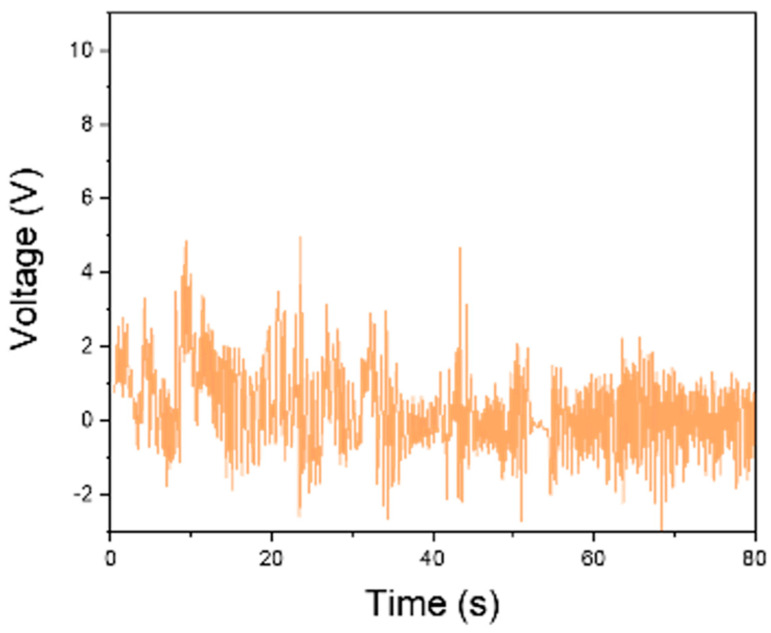
Voltage output of the triboelectric generator fabricated using drop-casting. The power generated using this system was in the range of 4.9 µW cm^−2^.

The ability of this device to self-power through triboelectricity (second mode) was studied. This energy generation was made possible due to the PDMS protective coating, which is charged positively in terms of triboelectric generators [66], since it can receive electrons without requiring a high energy. On the contrary, cellulose and lignin, the main components of leaves, are charged negatively due to the highly oxidised molecular structure, which allows the transfer of electrons to PDMS [66,67]. For the testing of the triboelectric properties of the films, a pristine tomato leaf was put into contact with a modified leaf, with the sensing device incorporated, and the leaves were moved against each other at a frequency of 1 Hz. A maximum voltage of 10 V and a current of 0.72 µA were obtained, which led to a power output of 7.2 µW cm^−2^ (Figure 4a). This value was higher than previously reported values for modified tree leaves [68], and even devices that incorporate more expensive materials, such as bacterial cellulose with ZnO nanoparticles [69], or BaTiO_3_ particles [70]. When the drop-cast sensor was employed for sensing, a lower power output of 4.9 µW cm^−2^ was obtained (Figure A14), which was a consequence of the lower surface area. As such, the aerosol-based device showed promise for the development of self-powered sensors for field applications. When no PDMS protective coating was applied to the leaf electrodes, the power output of the devices dropped below 0.3 µW cm^−2^ (Figure 4b), evidencing the need for a PDMS coating for the use of the electrodes in power generation applications.

**Figure A15:**
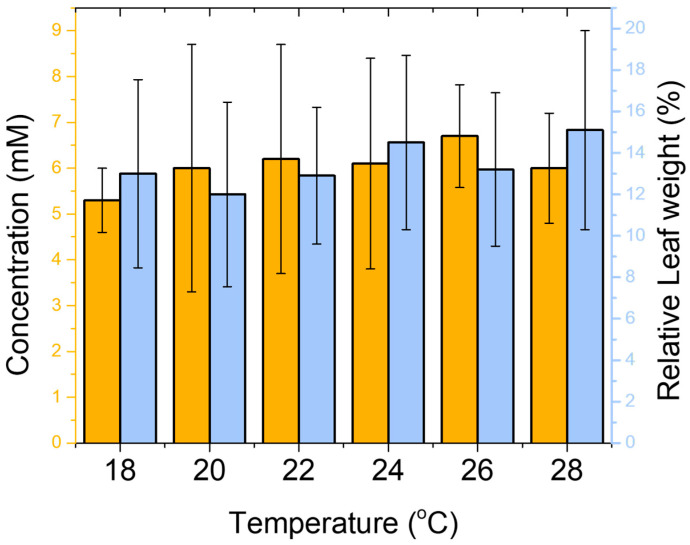
Sensor performance evaluated on a tomato leaf across a temperature range of 18 °C to 28 °C. The impedance measurements remain stable and show similar values across the range, indicating that environmental temperature has minimal interference with the sensor’s readings.

The sensing performance of the miniaturised sensors was also characterised. Initially, the effects of interferences due to environmental factors such as humidity and temperature were studied (Figure 4c and Figure A15). This is relevant in applications of this sensor as there are large differences in humidity and temperatures between the day and night. As such, it is desirable to minimise any interference with sensor behaviour due to this parameter. However, the sensitivity of PEDOT:PSS films to environmental humidity and temperature is well-documented [71,72]. While this can be a potential source of variability in measurements, a high degree of signal stability was observed under realistic plant growth conditions. During initial tests, the impedance response of the leaf sensor was measured under relative humidity values between 50 and 90%, where only small variations were recorded. A noticeable decline in impedance occurred only at 100% RH, which was attributed to condensation forming on the leaf surface rather than an intrinsic conductivity change in the PEDOT:PSS film. Measurements recorded under different temperatures (18–28 °C) were also stable, confirming that the hybrid PEDOT:PSS/PDMS electrodes remain robust even under varying environmental parameters. Slight deviations were occasionally observed, likely reflecting natural physiological adjustments of the plant to these conditions, such as stomatal conductance changes, but overall, the performance remained consistent. This stability was attributed to the leaf’s ability to maintain a relatively constant microenvironment. The combination of the boundary layer, stomatal regulation, and water transpiration creates a microclimate at the leaf surface that buffers against ambient humidity and temperature fluctuations [13].

**Figure A16:**
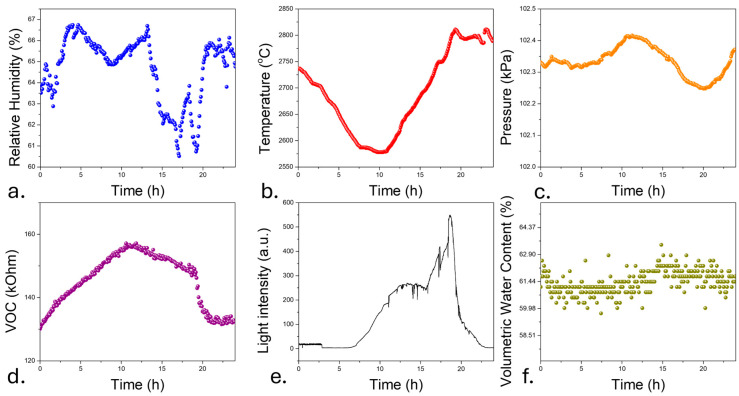
Environmental conditions in the controlled greenhouse during the 24 h monitoring experiment. Plots showing (**a**) relative humidity (%), (**b**) temperature (°C), (**c**) atmospheric pressure (kPa), (**d**) VOC concentration (kOhm), (**e**) light intensity, and (**f**) volumetric water content of soil (%). All parameters were recorded at 10 min intervals to ensure stable and reproducible conditions.

An in vivo test was then conducted by continuously measuring the ionic concentration and relative leaf weight on a plant for 24 h (Figure 4d). A decrease in the concentration, followed by a lower water loss, was measured during the nighttime compared to the light hours. These concentration values varied from approximately 5 mM up to 20 mM during the daytime. Similarly, the relative leaf weight increased by 15% during the day to down to 6% at night. The observed increase in ionic concentration during the light period, followed by higher leaf water loss, reflects the expected increase in transpiration and stomatal opening under photosynthetic activity. Conversely, the reduction in both ionic strength and water loss at night suggests a downregulation of stomatal conductance and reduced xylem flow. The ability to capture these dynamic oscillations in real time highlights the role of ion fluxes as a proxy for plant water use efficiency, which is difficult to obtain with conventional destructive assays. Environmental parameters were continuously recorded during the day–night impedance monitoring experiments to ensure stable and reproducible conditions. Measurements were performed in a controlled greenhouse environment equipped with a BME680 environmental sensor interfaced with a Wio Terminal data logger. The system simultaneously recorded temperature, relative humidity, atmospheric pressure, VOC concentration, and light intensity at 10 min intervals (Figure A16a–f). Soil moisture was monitored using a capacitive humidity probe inserted near the plant’s root zone. The probe was calibrated using soil samples with known gravimetric water contents to relate voltage output to relative field capacity. During the 24 h monitoring period, environmental conditions remained relatively stable, with temperature varying between 25.5 °C and 28.0 °C, humidity between 60.3% and 66.5%, and light intensity from 0 lux (night) to approximately 750 lux (day). Soil moisture fluctuated modestly between 61 and 69% field capacity, while atmospheric pressure (1004–1009 hPa) and the VOC index (55–63) showed a negligible impact on the impedance response. These environmental parameters confirm that the observed diurnal variations in impedance are mainly attributable to physiological changes in transpiration and ionic redistribution within the leaf, rather than to exogenous variations in temperature or humidity.

The performance of the sensors in the short term under a dynamic environment was demonstrated. The tomato plant was subjected to a highly saline solution. After 1 h, the calculated ionic strength increased, from 27 mM under standard conditions to up to 34 mM. On the contrary, the relative leaf weight did not experience a significant change, with it in the range of 18.5% (Figure 4e,f). As expected, both the ionic strength and relative leaf weight decreased when the plants were subjected to DI water with no ions.

**Figure A17:**
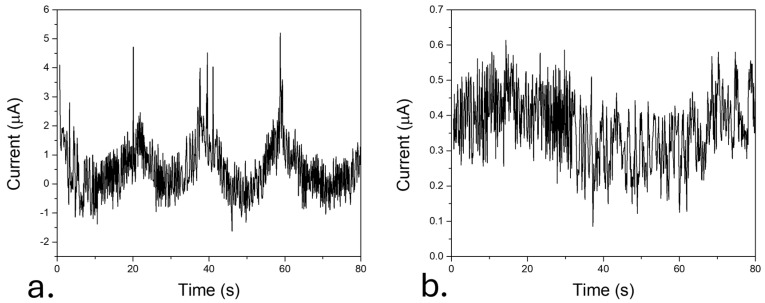
(**a**). The measurement shows the short-circuit output from the PEDOT:PSS/PDMS-coated tomato leaf (deposited by aerosol deposition), recorded under a 1 Hz contact–separation frequency. (**b**). The measurement was taken from a control setup (lacking the PEDOT:PSS and PDMS layers) under the same 1 Hz contact–separation frequency, showing negligible output.

Figure 4a shows the characteristic open-circuit voltage of the PEDOT:PSS/PDMS–leaf hybrid triboelectric device under a contact–separation frequency of 1 Hz (short-circuit current is shown on Figure A17a,b). A detailed look at the open-circuit voltage reveals an asymmetrical AC signal, where the positive voltage peaks are of a significantly greater magnitude than the negative peaks. This asymmetry is commonly observed in triboelectric nanogenerators, particularly those operating with non-uniform, manual mechanical motion [40]. The disparity in peak heights is primarily attributed to the inherent asymmetry in the mechanical cycle of the device [73]. Specifically, the manual contact–separation motion used to generate the signal does not have a perfectly uniform velocity. The speed of the “contact” stroke is typically different from the “separation” stroke. Since the TENG output is highly dependent on the rate of charge separation (i.e., the speed of motion), this velocity difference results in unequal positive and negative peaks [40]. Furthermore, the physical interaction during contact differs from that during separation. Interfacial adhesion forces (such as van der Waals forces or micro-suction from the leaf’s complex topography) can cause the two surfaces to “stick” slightly. This suggests that the separation phase is a “peeling” process, which is mechanically and dynamically different from the initial “contact” stroke, further contributing to the asymmetrical electrical output [73]. Similar behaviours have been observed in other leaf-based triboelectric generators [74].

The current waveform exhibited a reproducible periodic profile, from which the transferred charge per cycle was determined by numerical integration according to Equation (1). Based on these results, the average transferred charge was $5.14\times 10^{-6}C$ per cycle. Using Equation (2) and the geometric electrode area (2 cm^2^), the effective areal charge was calculated to be $25.7\;mC\;m^{-2}$, leading to an effective device capacitance of 5144 nF. The obtained surface charge density lies towards the upper end typically reported for flexible polymer- and nanomaterial-based triboelectric nanogenerators, including those developed using natural leaves, as shown in Table 1. These results were attributed to an effective charge transfer and strong conformal contact between the PEDOT:PSS/PDMS–leaf hybrid triboelectric device and rough leaf surfaces. This was reflected in a high apparent capacitance, which indicates an efficient charge storage within the hybrid interface.

Upon exposing the plant to DI water, both the relative leaf weight and the ionic strength decreased, to 16.5% and 20 mM, respectively. Such a change was a consequence of the deionised composition of DI water, which decreased the ionic concentration in the leaves. These results demonstrated the applicability of the PEDOT:PSS impedimetric sensors in the monitoring of the plant health status, which could be used to determine the water and electrolyte needs in real time. The short-term perturbation experiments further demonstrate physiological specificity. The rise in ionic strength after saline irrigation indicates the sensor’s ability to detect acute osmotic stress, while the decrease following DI water exposure reflects nutrient dilution within the apoplast. Importantly, the absence of major changes in leaf water content under these conditions indicates that ionic and hydraulic responses can be deconvoluted with this method. This separation is key, since conventional plant stress assays often conflate the two [79,80].

The observed cyclic impedance fluctuations align with the plant’s day–night transpiration patterns, as stomatal opening during the photoperiod increases ionic exchange and hydration at the epidermis [81]. During nutrient depletion under deionised water irrigation, reduced ionic strength in the apoplast leads to a measurable rise in resistance and a decline in capacitance, mirroring early physiological stress. Similar relationships between ionic composition, hydration, and impedance have been reported in plant bioelectronic systems [17,82].

**Figure A18:**
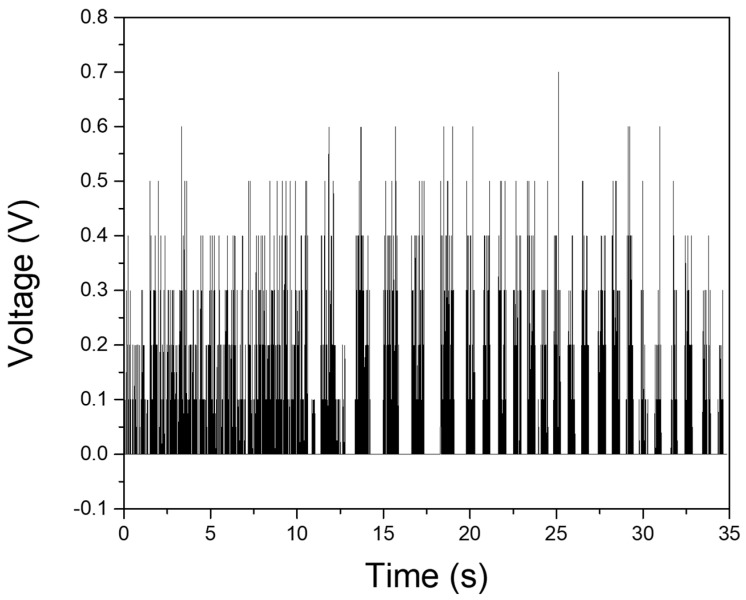
Open-circuit voltage (Voc) of the hybrid triboelectric device.

The triboelectric response of the leaf tattoo device was further examined under simulated rainfall conditions by subjecting sensor-coated leaves to a continuous stream of water (150 mL min^−1^) directed onto the leaf surface on an intermittent patter: 1 s on and 1 s off (no flow), for a period of 30 s (Figure A18). The recorded output voltage was considerably smaller compared with those obtained during mechanical leaf–leaf contact (<1 V), indicating that charge generation through liquid–solid interaction was minimal. This behaviour is consistent with the hydrophobic nature of the PDMS encapsulation layer, which restricts wetting and charge exchange with water droplets.

Water contact angle measurements confirmed this interpretation, yielding a mean contact angle of 103 ± 2°, indicative of poor wettability and limited surface interaction. Consequently, the triboelectric output under water exposure was less than 0.2 µW cm^−2^, demonstrating that the device is effectively immune to electrical artefacts caused by irrigation or rainfall.

### 3.4. Long-Term Monitoring of Sensors

**Figure 5:**
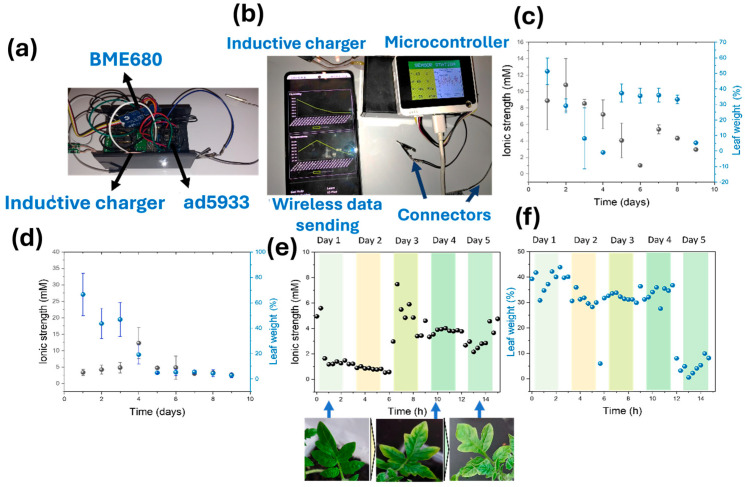
(**a**). Final device embodiment employed for the determination of the impedance on plant leaves. The device contains an ad5933 system, a bme680 for environmental monitoring, and inductive chargers connected to a Wio Terminal. This design allowed the determination of environmental and plant parameters. (**b**). The use of inductive chargers allowed a battery-free operation of the system, which could also send the data wirelessly to online servers (i.e., Adafruit IO) for IoT applications. (**c**). Ionic strength and leaf-water-loss values for plant A, irrigated with deionised water for four days followed by fertiliser supplementation on Day 6. (**d**). Ionic strength and leaf weight of a plant watered with DI water for 9 days. (**e**). Results obtained during the daily monitoring of the ionic strength of tomato plants watered with DI water for 5 days. The changes in leaf colouration due to the lack of ions are shown. (**f**). Results obtained from the weight measurement of the same plant.

One of the potential applications of the sensing device reported here is the monitoring of water and fertiliser needs of plants in the long term. The high stability and the biocompatibility of sensing films allowed the long-term monitoring of leaf ionic strength and water content. As a proof of concept, we deposited a PEDOT:PSS electrode onto a 6-week-old tomato plant, and we assembled a microcontroller device incorporating an ad5933 impedance analyser. The final system incorporated an inductive charger to avoid the need for batteries and could send the data wirelessly for IoT applications (Figure 5a). In addition, a bme680 was incorporated to allow the measurement of key environmental parameters (temperature, humidity, and VOC concentrations). As such, the device could be charged directly by employing a suitable mobile phone (Figure 5b), and it could generate power through triboelectricity. In this work, the triboelectric generator was implemented as a proof-of-concept demonstration to show that the PEDOT:PSS/PDMS leaf tattoo electrodes can harvest mechanical energy from natural leaf–leaf interactions. For testing of sensing capabilities of the devices, an inductive charging module was used, which ensured stable operation during long-term monitoring. However, the measured triboelectric output (7.2 µW cm^−2^) is sufficient to support future autonomous designs. By coupling the leaf-based generator to a small energy storage element (i.e., a flexible supercapacitor or thin-film microbattery) and a low-power charge management circuit, harvested energy could drive intermittently triggered sensing cycles or burst-mode wireless transmission.

Both the capacitance and charge transfer resistance of the leaves were measured during the daytime for 9 days, and the results were correlated with the ionic strength and water loss rates. Two plants were studied to demonstrate the application of the device in health monitoring. Both plants were initially watered using DI water every 2 days. After 4 days, one of the plants was supplemented with 5 mL of DI water containing 10 µL of fertiliser, while the second plant was watered using DI water only throughout the entire experiment (Figure 5c,d). The composition listed in Table A1 corresponds to the guaranteed nutrient analysis provided by the manufacturer (Hygeia Chemicals Ltd., Oranmore, Ireland). As is standard for commercial liquid fertilisers, the reported nutrient values do not sum to 100% because they represent only the concentrations of active nutrients. To generate the long-term trend data, impedance measurements were recorded daily within a 2 h window (at 20 min intervals) under stable daytime conditions. Figure 5c,d show the summary of this 9-day monitoring period for the fertilised and control (DI water only) plants, respectively. To provide a more detailed view of the nutrient depletion, Figure 5e,f show the daily measurements of ionic strength and leaf weight loss, respectively, for the control plant during the first 5 days. The accompanying photographs in Figure 5e visually document the onset of chlorosis (yellowing) observed during this period.

Results showed a consistent decrease in the ionic strength of the plant leaves over time, due to the use of DI water, which does not contain any ions (Figure 5d,e). In the case of the plant watered with DI water alone, the ionic strength value reached 2.71 mM (Figure 5d). For the fertilised tomato plant, a minimum concentration of 0.56 mM was measured on Day 6 just before supplementation, down from an initial 8.9 mM. This low value in concentration could potentially decrease the production yield of the tomatoes due to the lack of nutrients. In addition, the colouration of the plant leaves in both cases turned yellow, which is known as chlorosis and is indicative of poor health (Figure 5e, inset photos). Upon fertilisation on Day 6, the ionic strength initially rose to approximately 1.02 mM immediately post-watering and further recovered to 5.48 mM on the following day (Day 7) (Figure 5c). This recovery in ionic strength resulted in a concentration closer to the initial physiological values, and the yellowing of leaves decreased, showing an improvement in health status. In contrast to the ionic strength, the leaf water loss did not remain constant but exhibited marked fluctuations throughout the observation period. For instance, the relative leaf weight loss spiked from 1.01% on Day 4 to 36.58% on Day 5, and later dropped from 33.02% on Day 8 to 5.48% on Day 9 (Figure 5c). These sharp variations reflect dynamic physiological adjustments, such as changes in stomatal conductance, distinct from the progressive depletion observed in ionic strength.

These long-term measurements provide an additional layer of physiological insights. The gradual decline in ionic strength under repeated doses of DI water reflects a progressive nutrient depletion, which manifested as chlorosis. This trajectory mirrors the onset of nutrient deficiency syndromes that typically require days to weeks to become visually apparent. The recovery of ionic strength to near-basal values after fertiliser supplementation indicates that the impedance signal directly tracks nutrient resupply to the leaves. The leaf water loss profile remained relatively stable despite substantial shifts in ionic content, demonstrating that the water status and ion balance are partially uncoupled under mild nutrient stress, a low-ionic-strength condition in which insufficient nutrient availability lowers ionic conductance but does not yet generate appreciable changes in hydration. This behaviour is fundamentally different from osmotic stress, which occurs under high ionic strength and drives water efflux due to decreased leaf water potential. The ability of our tattoo sensor to distinguish these regimes highlights its sensitivity to early-stage, sub-visual nutrient stress before irreversible morphological symptoms arise.

## 4. Conclusions

This work reports a design for a non-invasive device for the monitoring of water needs and ion concentrations in plants. This sensor consisted of a PEDOT:PSS thin film deposited onto a tomato leaf using a low-cost aerosol-based method. A methacrylate mask patterned using laser cutting was incorporated, and nanometrically thin films could be deposited, as shown by a stylus profilometer. Devices were then protected using a PDMS coating. The final system could determine both the ionic strength and water loss of the leaves through the measurement of surface impedance using a low-cost microcontroller device with high resilience against environmental conditions (i.e., humidity, temperature, rainfall). Through the calibration using NaCl electrolytes, a limit of detection within the millimolar level was achieved. This performance allowed the non-invasive monitoring of electrolytes in plants. In addition, the presence of PDMS within the sensor structure allowed the generation of electrical power, which could enable a future battery-free embodiment. The generated power was in the range of 7.2 µW cm^−2^, higher than some of the more expensive cellulose-based approaches. To achieve fully autonomous sensing and wireless transmission without external charging, the harvested power must meet the hourly energy demands of electronics. While our device requires an energy supply in the order of milliwatts (mW), ultra-low-power sensors with a consumption within the µW range and lower have been reported [83,84]. The current output of 7.2 µW cm^−2^ places our system close to this threshold. Full autonomy could be achieved by slightly increasing the effective harvesting area and coupling the generator to a small on-leaf energy storage element like a thin-film battery, capable of accumulating charge throughout the day.

As a proof of concept, a device was deposited onto a plant leaf. The effect of humidity on the signal measurement were first determined. In this case, a decrease in measurements was only observed when the sensors were subjected to high humidity, close to 100 RH%. This high humidity resilience could enable the use of this device in field applications. The changes in the electrolyte concentrations upon watering with a 0.1 M NaCl concentration and DI water were then recorded. The observed changes in ionic strength and water loss were consistent with the added solution, with a higher ion concentration when water containing high salt concentrations was applied (34 mM) compared to the initial value (27 mM), and a lower concentration when DI water was added (20 mM). On the contrary, when only DI was added, the water content of the leaves increased. This was attributed to the osmotic pressure taking place due to the absence of ions within the solution used.

The long-term performance of the sensors deposited onto the leaves was also demonstrated. In this case, the employed microcontroller was incorporated using an inductive charger to avoid the use of batteries, and an intuitive interface that allowed the wireless reporting of data was programmed. This device incorporated an environmental sensor for the determination of temperature, humidity, and VOC concentration. The ionic strength and water content of the leaves could be recorded for 9 days, and the plants were watered with DI water. The lack of electrolytes in the water was reflected in the impedance plots, proving the applicability of our sensing device in the early detection of plant stress due to the environmental conditions. Upon watering the plant with a fertiliser supplement on Day 6, the ionic strength of the leaf recovered from a minimum of 0.56 mM to 5.48 mM on the following day, returning to similar levels as prior to the use of DI water. These results represent a step forward in the field of wearable sensing in plants, which could be exploited in the future development of wireless and self-powered biosensors, with a plethora of applications in smart agriculture and environmental monitoring, among others.

## Figures and Tables

**Table 1:** Comparison of triboelectric generator performance, compared to reported values in the literature for similar devices.

	$V_{OC}(V)$	$I_{SC}(\mu A)$	$Q(C\;cycle^{-1})$	$\sigma_{eff}=Q/A(mC\;m^{-2})$	$\mathrm{Power}\;\mathrm{Density}\;(W\;m^{-2})$	
PEDOT:PSS/PDMS—tomato leaf	~10	5.14	5.14 × 10^−6^	25.7	0.072	This work
Natural-leaf-based single-electrode TENG	12	3.8	4.2 × 10^−6^	16.8	0.056	[75]
Bamboo-leaf TENG	~191	~5.0	-	-	-	[76]
Nitro/Methyl-CNF TENG	8	9	-	-	-	[77]
Bacterial-cellulose/ZnO hybrid bio-TENG	58	5.8	-	-	0.042	[69]
PDMS doped with carbon black and polyvinylpyrrolidone	50	5.5	6.2 × 10^−7^	3.1	0.45	[78]

## Data Availability

The original contributions presented in this study are included in the article. Further inquiries can be directed to the corresponding author.

## Appendix A

The characteristic Voc generated by the PEDOT:PSS/PDMS-coated tomato leaf, recorded under a 1 Hz contact–separation frequency.

**Table A1:** Guaranteed nutrient analysis of the commercial liquid fertiliser (Hygeia Chemicals Ltd., Oranmore, Ireland) used in this study. Only the nutrient values disclosed by the manufacturer are shown; the remaining mass consists of water and proprietary formulation components not listed on the product’s technical sheet.

Compound	Amount (wt%)
Total Nitrogen (N)	6
Nitric Nitrogen (N)	1.2
Ureic Nitrogen (N)	4.8
Phosphorous Pentoxide	5
Potassium Oxide	9
Magnesium	0.02
